# Single-molecule visualization of a formin-capping protein ‘decision complex' at the actin filament barbed end

**DOI:** 10.1038/ncomms9707

**Published:** 2015-11-13

**Authors:** Jeffrey P. Bombardier, Julian A. Eskin, Richa Jaiswal, Ivan R. Corrêa, Ming-Qun Xu, Bruce L. Goode, Jeff Gelles

**Affiliations:** 1 Department of Biochemistry, Brandeis University, Waltham, Massachusetts 02454-9110, USA; 2 Department of Biology, Brandeis University, Waltham, Massachusetts 02454-9110 USA; 3 New England Biolabs, Ipswich, Massachusetts 01938, USA

## Abstract

Precise control of actin filament length is essential to many cellular processes. Formins processively elongate filaments, whereas capping protein (CP) binds to barbed ends and arrests polymerization. While genetic and biochemical evidence has indicated that these two proteins function antagonistically, the mechanism underlying the antagonism has remained unresolved. Here we use multi-wavelength single-molecule fluorescence microscopy to observe the fully reversible formation of a long-lived ‘decision complex' in which a CP dimer and a dimer of the formin mDia1 simultaneously bind the barbed end. Further, mDia1 displaced from the barbed end by CP can randomly slide along the filament and later return to the barbed end to re-form the complex. Quantitative kinetic analysis reveals that the CP-mDia1 antagonism that we observe *in vitro* occurs through the decision complex. Our observations suggest new molecular mechanisms for the control of actin filament length and for the capture of filament barbed ends in cells.

## Abstract (web summary)

Formins promote the polymerization of actin filaments at barbed ends and capping protein prevents polymerization. Here the authors use single molecule imaging to directly visualize a decision complex consisting of the formin mDia1 and capping protein bound simultaneously to the filament barbed end and the dynamic mechanisms by which it forms and dissociates.

## Graphical abstract

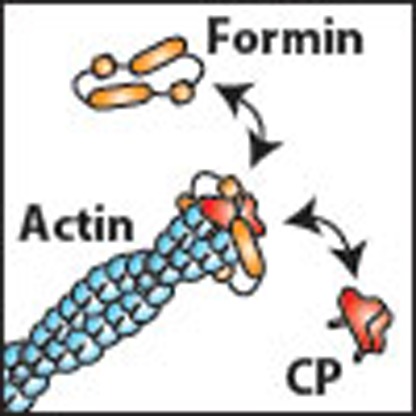

Actin filaments form architecturally and functionally diverse structures inside cells, and many of these structures have characteristic filament lengths^1^. It is clear that the polymerization of actin into filamentous networks is tightly regulated by numerous actin-associated proteins, some with seemingly antagonistic activities. In some cases cooperation among these proteins can provide new levels of regulatory control over the construction of actin networks^2^.

One factor that contributes to limiting actin filament elongation and thus length in cells is capping protein (CP). CP is present at μM concentrations in most cells, comparable to the concentration of filament barbed ends, and binds with high affinity to barbed ends (100 pM) and arrests dynamics (reviewed in ref. 3). As such, CP is a key component in the dendritic actin assembly model of lamellipodial-based cellular locomotion, and is necessary for a wide range of cellular and physiological processes that depend on actin dynamics^3^,^4^. In addition, CP is a prominent component of the Z lines of sarcomeres, where it helps to capture and anchor filament barbed ends. Interestingly, at least four different formins (see below) have also been localized to the Z lines^5^,^6^,^7^.

The dynamics-arresting action of CP can be counteracted by barbed end-associated proteins such as formins and Ena/VASP, which at once prevent CP binding while still allowing elongation^2^,^8^,^9^,^10^. Among the 15 different formins expressed in mammals, mDia1 is one of the most well-studied and is notable for the very fast rates of filament polymerization it facilitates, accelerating elongation by at least fivefold in the presence of profilin^11^,^12^. Single-molecule fluorescence experiments show that a single formin molecule can remain associated with the barbed end for minutes while it grows^13^,^14^. This persistent association by formins with the barbed ends of elongating filaments has led to the view that formins protect growing filament ends from CP, thus allowing growth even at the high concentrations of CP possible in cells^15^,^16^,^17^,^18^. Further, this view is supported by genetic observations suggesting that CP and formins counterbalance each other's activities *in vivo*^17^,^19^,^20^. Until now, the mechanism underlying this antagonism between formins and CP, and precisely how it influences actin filament dynamics, has gone unresolved. Most biochemical evidence to date has been interpreted to suggest that a formin must dissociate from the barbed end of a filament before CP can bind and cap the filament^15^,^16^,^18^, but alternative interpretations are possible.

Here we labelled mDia1 and CP each with a different colour fluorescent dye and used multi-wavelength single-molecule fluorescence methods^21^,^22^ to directly and simultaneously observe the dynamic interactions of the labelled proteins with individual actin filament barbed ends. The antagonistic regulation of barbed-end growth by CP and mDia1 occurred exclusively through the reversible formation of a long-lived ‘decision complex' intermediate, in which CP and mDia1 were simultaneously present at the barbed end. Through the decision complex, CP could displace mDia1 from the barbed end. Further, displaced Dia1 could randomly slide along the actin filament and could later re-form the decision complex, in some cases leading to CP displacement and resumed barbed-end growth. Taken together, our results quantitatively define the kinetic mechanism of mDia1/CP associative competition and give new insights into how actin filament length and positioning may be controlled *in vivo*.

## Results

### CP arrest of mDia1-mediated actin polymerization

**Figure 1:**
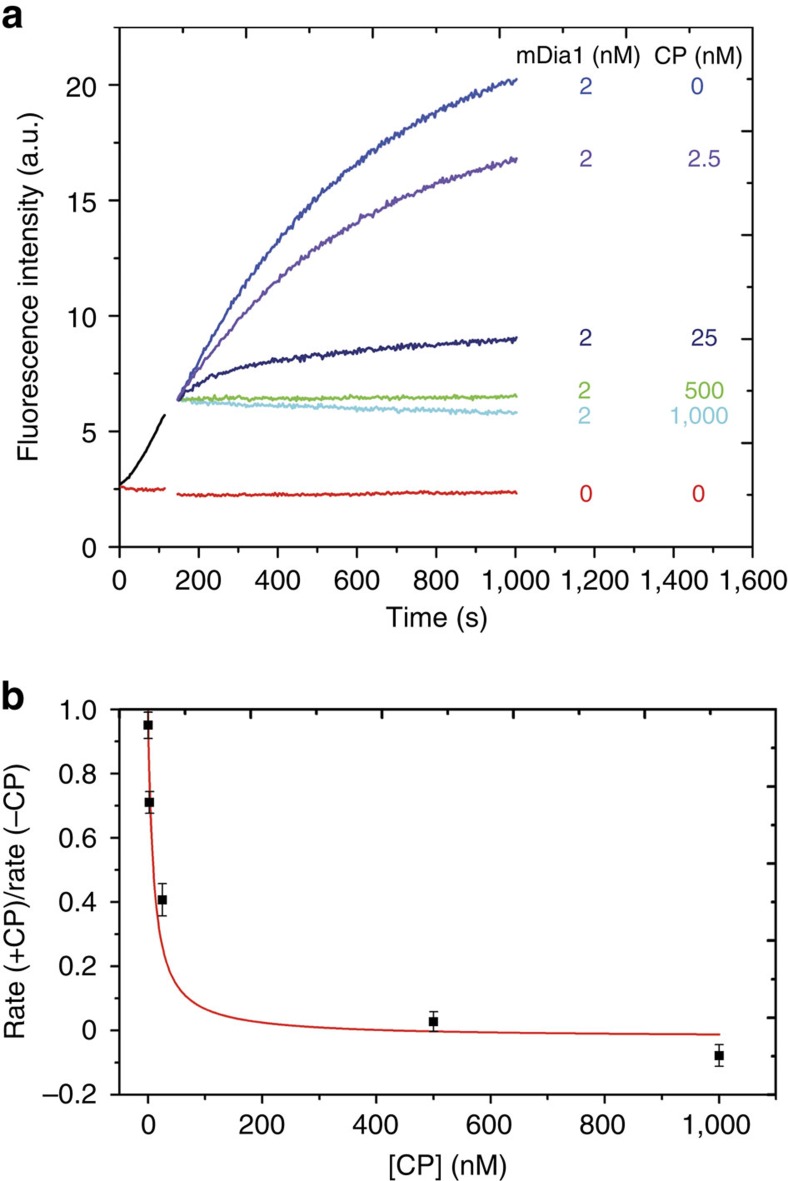
mDia1-mediated actin assembly is antagonized by introduction of CP. (**a**) Assembly of 2 μM actin monomers (10% pyrene labelled) was initiated in the presence of 5 μM profilin by the addition of the indicated concentration of mDia1 and monitored by pyrene fluorescence enhancement. At 113 s CP at the indicated concentration was added to the reaction. For clarity of presentation, assembly curves before CP addition were averaged (black), and curves after CP addition were vertically offset to start at a common point. (**b**) Ratio (±s.e.) of actin polymerization rates over 40 s just before and over 40 s just after CP addition measured from the individual curves in **a** (points) and polynomial fit (line) yielding IC_50_=10±4 nM (s.e.). Data are derived from a single experiment.

To begin investigating the mechanism of antagonism between CP and formins, we asked whether CP interferes with the elongation of mDia1-capped filaments by monitoring the rate of pyrene–actin incorporation into filaments previously nucleated by mDia1. The mDia1 construct used contains the formin homology 1 (FH1) domain through the C terminus and is constitutively active for filament nucleation and processive elongation^14^. In the presence of profilin, nucleation is almost completely mDia1 dependent (Fig. 1a, 0–100 s, red versus black curve), and essentially all filaments formed under these conditions are expected to retain mDia1 at their ends^12^,^23^. When CP was added to the growing filaments at a later time point, the rate of polymerization declined (Fig. 1a, 125–1,000 s), with both the magnitude and the rate of the decline increasing at increasing CP concentrations. These data suggest that CP can arrest the polymerization of filaments previously initiated by mDia1.

Only ∼0.1 nM CP is required to inhibit polymerization or depolymerization of uncomplexed actin filaments^24^,^25^. By contrast, the concentration of CP required in our experiments for half-maximal inhibition of the growth of formin-initiated filaments was roughly 100-fold higher (Fig. 1b). This is consistent with previous proposals^16^,^17^,^18^,^26^ that formins protect barbed ends from CP, but suggests that this protection is only partial, in that growth of filaments nucleated and elongated by formins is stopped at high CP concentrations.

### Oligomeric state of mDia1 on growing filament ends

To further elucidate the mechanism of CP/mDia1 antagonism, we first defined the oligomeric state of mDia1 as it processively elongates a growing filament. mDia1 is a dimer in solution before it binds to filaments^2^,^14^,^15^,^16^ and it has been hypothesized to retain a dimeric state on filament ends^2^,^13^,^15^,^27^,^28^, but that has never been demonstrated.

**Figure 2:**
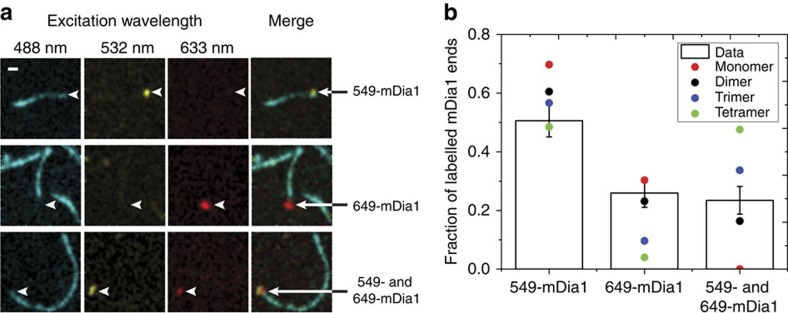
Three-colour TIRF imaging of the barbed ends of actin filaments elongating in the presence of dual-labeled mDia1. Filaments were grown from 1 μM G-actin (10% labelled with the blue-excited dye AF488) in the presence of 3 μM profilin plus an mDia1 preparation containing a mixture of green-excited (549-mDia1) and red-excited (649-mDia1) fluorescent subunits (*N*=107 barbed ends with fluorescent formins observed; 0.39 and 0.17 mol dye per mol subunit for 549-mDia and 649-mDia1, respectively). (**a**) Three examples of filament barbed ends (marked by arrowheads) that exhibited only 649-mDia1 fluorescence (top row), only 549-mDia1 fluorescence (middle row) or both (bottom row). In the merged images here and in subsequent figures, the 549 and 649 images are slightly offset so that a single molecule labelled with both dyes is imaged as adjoining red and yellow spots. Scale bar, 2 μm. (**b**) Observed fractions of fluorescently labelled barbed ends with only 549-mDia1, only 649-mDia1 or both (bars; ±s.e.) and fractions predicted by models in which the mDia1 catalysing the growth of the barbed end is exclusively in the indicated oligomeric state (points). Models were based on independently measured labelling stoichiometries and have no free parameters.

We characterized the oligomeric state of mDia1 on growing filament end in three-colour single-molecule fluorescence experiments. We first prepared SNAP-mDia1, a SNAP-tagged^29^ construct that is a dimer in solution like native mDia1 and assembles filaments *in vitro* with kinetics essentially identical to the corresponding mDia1 fragment^14^. We reacted the SNAP-mDia1 with an equimolar mixture of the benzylguanine adducts of green-excited and red-excited fluorescent dyes, yielding a statistical mixture of green (549-mDia1) and red (649-mDia1) subunits in the formin dimers. On incubation with blue-excited AF488 actin, the dual-labelled formin preparation was observed in total internal reflection fluorescence (TIRF) microscopy to nucleate actin filaments that grew while fluorescent formin molecules remained processively associated with their barbed ends (Fig. 2a and Supplementary Movie 1). In addition to barbed ends containing only red or only green formin subunits, some ends with both red and green were observed (Fig. 2b), suggesting that these ends were being elongated by a molecule containing at least two mDia1 subunits. The colours present at an end did not change during observation (typically ∼1 min per end) suggesting that formin subunits did not exchange with others from solution at the sub-nM concentrations used. Counts of the numbers of growing filament ends with fluorescence from only red, only green or both red and green subunits were far more consistent with the hypothesis that a formin dimer is the active species that catalyses elongation than they were with monomer, trimer or tetramer models (Fig. 2b). The presence of a kinetically stable formin dimer at the barbed end is consistent with proposed dimer ‘stair-stepping' models for formin function in actin filament elongation^15^,^27^.

### Formation of an mDia1/CP/barbed-end complex

Next, we proceeded to use the same three-colour TIRF methodology to observe the interactions of filament ends with single molecules of both mDia1 and CP. For these experiments, we purified CP heterodimer with a SNAP tag fused to the N terminus of the β2 subunit and fluorescently labelled it. The resultant 649-CP, like wild-type CP, quickly arrests actin filament elongation by binding to barbed ends (Supplementary Fig. 1).

To simultaneously monitor mDia1 and CP interactions with filament barbed ends, actin filaments were grown in the presence of 50 pM 549-mDia1 and 3 μM profilin in the presence or absence of 649-CP. As we previously reported^14^, in the absence of CP single molecules of mDia1 remained associated with the barbed ends as filament growth continued (Supplementary Movie 2). Typically, filaments were observed to grow continuously until they exited the microscope field of view. In contrast, when 649-CP (at concentrations of 3–12 nM) was added to the reaction, we frequently observed filaments initially growing with 549-Dia1 at the barbed end and then being converted into non-growing filaments with 649-CP at the end (Supplementary Movies 3 and 4), consistent with the behaviour seen in bulk (Fig. 1).

**Figure 3:**
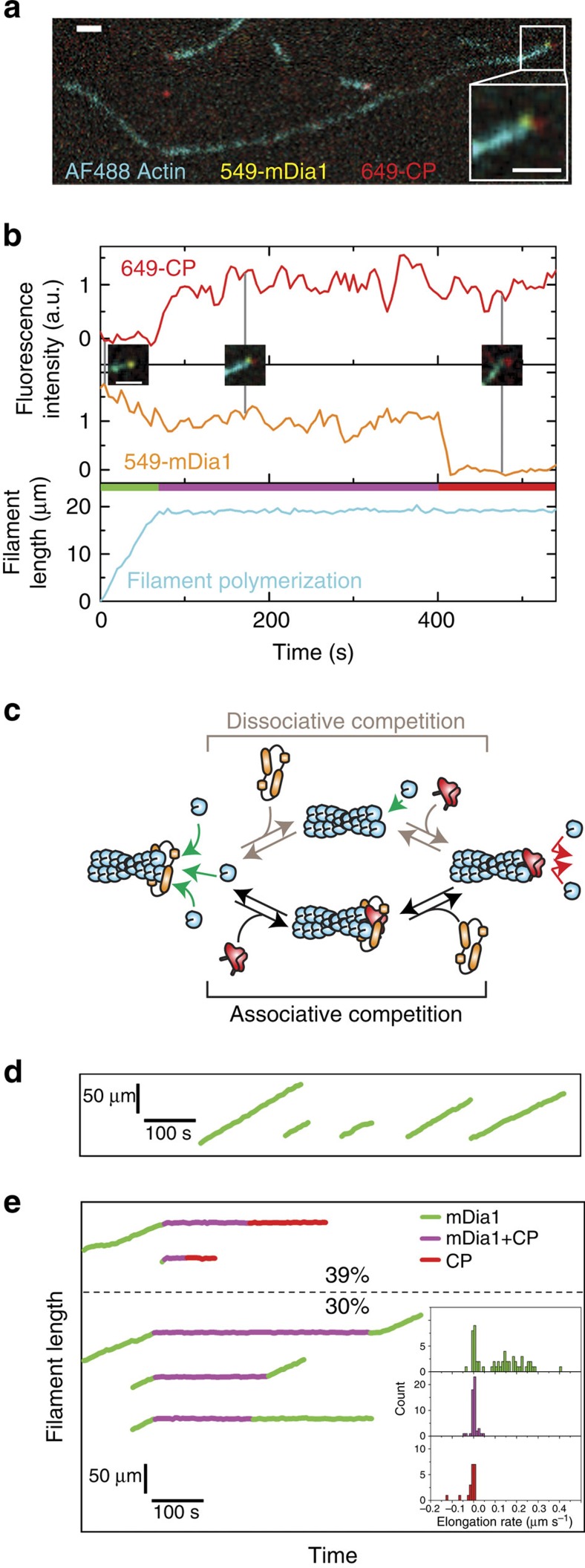
mDia1/CP/actin filament barbed-end complex (*N*=171 complexes observed). (**a**) Merged three-colour TIRF image of the actin filament (blue) in Supplementary Movie 3, showing 549-mDia1 (yellow) and 649-CP (red) molecules bound simultaneously to the barbed end. Inset: magnified and filtered view of the indicated barbed end. (**b**) Fluorescence intensity and filament length record of the filament in **a**. Colour ribbon indicates the time intervals in which the barbed end is occupied by 549-mDia1 only (green), 649-CP only (red) or both proteins (magenta). Insets: three-colour merged images of the barbed end using the same colour scheme as in **a**, taken at the indicated times. Scale bars in **a** and **b**, 2 μm. Frame interval: 5 s. (**c**) Possible mechanisms for CP antagonism of mDia1-catalysed filament elongation. mDia1 (orange) elongates actin filaments (blue) by incorporating actin subunits (green arrows). The dissociative pathway requires the formin to first dissociate before CP (red) can bind the barbed end; the associative pathway allows CP to bind a formin-occupied barbed end before formin dissociation. (**d**,**e**) Example filament length records from actin polymerization experiments containing 549-mDia1 alone (**d**) or 549-mDia1 plus 649-CP (**e**). Traces are coloured to indicate which proteins are visible at the barbed end, using the same colour scheme as in the ribbon in **b**. Percentages indicate the fraction of 549-mDia1/649-CP/barbed-end complexes (out of *N*=171) that ended by loss of 549-mDia1 fluorescence (top) or loss of 649-CP fluorescence (bottom). Percentages do not sum to 100 because some the dissociation of some complexes could not be unambiguously scored due to overlap with the images of other filaments. Inset: mean elongation rate in filament record segments classified by which barbed-end proteins were present. The experiments in this and all following figures used 0.5–1 μM actin in the presence of 1.5–3 μM profilin.

Significantly, the transition from a growing, mDia1-associated end to a non-growing CP-associated end proceeded through a characteristic sequence of events. We never (0% of *N*=40 mDia1-bound barbed ends) observed 549-mDia1 dissociation followed by continuing filament growth and subsequent 649-CP binding. Instead, we almost always (93%) saw that capping occurred through the formation of an unexpected long-lived ternary complex between 549-mDia1, 649-CP and the barbed end (Fig. 3a,b and Supplementary Movies 3 and 4). (In the remaining 7% of events, the transition of the barbed end from 549-mDia1 bound to 649-CP bound happened too quickly to discern the order of the dissociation and binding events.) The high proportion of events in which CP was observed to bind before departure of mDia1 from the barbed end suggested that CP usually or always acts on barbed ends being elongated by mDia1 through an ‘associative competition' mechanism (Fig. 3c, bottom), rather than the long-presumed dissociative mechanism (Fig. 3c, top). Differentiating between these two mechanisms is critical, because they have profoundly different implications for cellular function, including the duration of formin ‘runs' on filament ends, the regulation of filament length distribution in actin networks and the ability of localized CP to capture the barbed ends of growing filaments (see Discussion).

We next examined actin polymerization at barbed ends with bound mDia1 and CP. When only 549-mDia1 fluorescence was detected at the barbed end, most filaments grew at a constant rate with no appreciable pauses in elongation (Fig. 3d,e). Occasionally, a 549-mDia1-terminated filament was observed to grow continuously and then stop, possibly due to photodamage. In contrast, during periods in which both 549-mDia1 and 649-CP were observed on the barbed end, the filament growth rate was uniformly zero within experimental uncertainty (Fig. 3e), and as expected, barbed ends with 649-CP alone did not grow. Thus, bound CP halts subunit exchange at barbed ends whether or not mDia1 is simultaneously present. Similar results were obtained for another fluorescently labelled formin, human DAAM1 (Supplementary Fig. 2), showing that these phenomena are not confined to mDia1. Control experiments showed that formation of the mDia1/CP/barbed-end complex was not an artefact of protein tagging and/or labelling (Supplementary Fig. 3).

### Fates of the mDia1/CP/barbed-end complex

In somewhat less than half (39%; 67 of 171) of the mDia1/CP/barbed-end complexes, we observed the filament end eventually lose 549-mDia1 fluorescence, yielding a 649-CP-capped filament that never resumed growth (examples in Fig. 3b,e, top). Loss of fluorescence was typically abrupt (Fig. 3b at 400 s) and the lifetime of 549-mDia1 fluorescence in the complexes was largely invariant to changes in laser exposure of the 549 dye (Supplementary Fig. 4a). Thus, loss of fluorescence was caused in nearly all cases by dissociation of 549-mDia1 from the barbed end rather than by photobleaching.

For other complexes (30%; 51 of 171; Fig. 3e, bottom), the simultaneous residence of 549-mDia1 and 649-CP (at the barbed end) ended with the disappearance of the 649-CP spot. In 21 of a sample of 22 649-CP loss events analysed in detail, fluorescence disappeared in a single abrupt step (for example, Supplementary Fig. 5), and 549-mDia1 fluorescence duration in complexes was essentially independent of laser exposure (Supplementary Fig. 4b). Since CP was labelled on only one subunit, this suggests that the complex contains a single CP heterodimer and that fluorescence loss reports dissociation of the dimer from the complex rather than photobleaching. Most often (66%; 24 of 36 measurable), filament polymerization resumed immediately after 649-CP departure (for example, Supplementary Movie 5). In a minority of cases (34%) growth did not resume, possibly because of actin photodamage^30^. Taken together, our observations suggest that the mDia1/CP/barbed-end complex contains one mDia1 dimer and one CP dimer, and show that it is resolved by either mDia1 leaving, which yields a capped end, or CP leaving, which most often yields an mDia1-associated polymerizing end. We therefore term the complex the ‘decision complex' because it represents a decision point from which the filament either resumes growth or becomes conventionally capped.

### Sliding and recapture of mDia1

**Figure 4:**
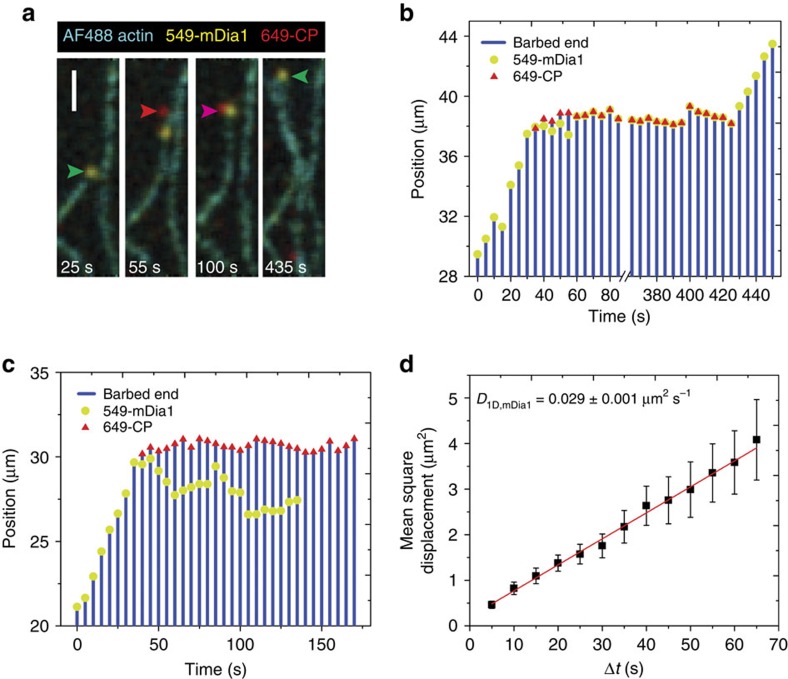
Sliding of mDia1 molecules along actin filaments in the presence of 649-CP (*N*=63 sliding events observed). (**a**) Merged three-colour TIRF images of the same field of view (from Supplementary Movie 6) taken at the indicated times. Initially, a 549-mDia1 molecule (green arrowhead) was observed elongating a filament. Subsequently, 649-CP (red arrowhead) bound the barbed end and displaced 549-mDia1, which slid along the filament. Later, 549-mDia1 recaptured the barbed end, re-forming the decision complex (magenta arrowhead). Finally, 649-CP dissociated, which permitted 549-mDia1-mediated elongation to restart. Scale bar, 2 μm. (**b**) Position records for barbed end, 549-mDia1 and 649-CP for the filament in **a**. (**c**) Same as **b**, for another example mDia-1 sliding event on a different filament. In this case mDia1 was not recaptured at the barbed end and the filament remained capped (*N*=11 recapture events observed). (**d**) The mean square displacement (±s.e.m.) between pairs of points separated by time interval Δ*t*, taken from the position records of 10 sliding 549-mDia1 molecules. A linear fit (red) yielded the diffusion coefficient *D*.

When 549-mDia1 molecules dissociated from the decision complex, they often disappeared in a time ≤ 0.5 s, the duration of a single video frame, consistent with release into solution and expected rapid diffusion out of the evanescent field of the TIRF microscope. However, in a significant fraction of the 549-mDia1 dissociation events (43% of *N*=63 measurable), the molecule did not disappear from the image, and instead was observed to slide along the filament (Fig. 4a–c and Supplementary Movie 6). Such sliding has not been observed previously for mDia1, but was reported for another formin, DAAM1 (ref. 31). The sliding motions were bidirectional, and had the characteristics of a one-dimensional random walk along the filament (Fig. 4d). The apparent diffusion coefficient (Fig. 4d) was ∼100-fold lower than that calculated from a barrier-less helical diffusion model (described in ref. 32 and based on a model in which mDia1 is centred at a radial position 7.7 nm from the filament axis), indicating that the observed motion is consistent with either helical or linear sliding and that there is a small but significant energy barrier for the sliding mDia1 molecule to move from one position to the next on the filament lattice.

Two different fates were observed for sliding 549-mDia1 molecules. In some cases, the sliding 549-mDia1 was observed to eventually disappear from the side of the filament due to dissociation (or possibly photochemical or chemical bleaching), leaving behind a filament with a CP-capped barbed end (Fig. 4c). In other cases, 549-mDia1 diffused back to the barbed end and re-formed the decision complex, which sometimes led to dissociation of 649-CP and resumed filament elongation (Fig. 4a,b). These observations clearly demonstrate that a substantial fraction of mDia1 molecules enter the sliding state on leaving the filament barbed end (the observed 43% is likely an underestimate of this fraction since very short sliding episodes would not be detected). The data also show that when present on a capped filament, a sliding mDia1 molecule can re-form the decision complex and reverse capping.

### Dynamics of associative competition

**Figure 5:**
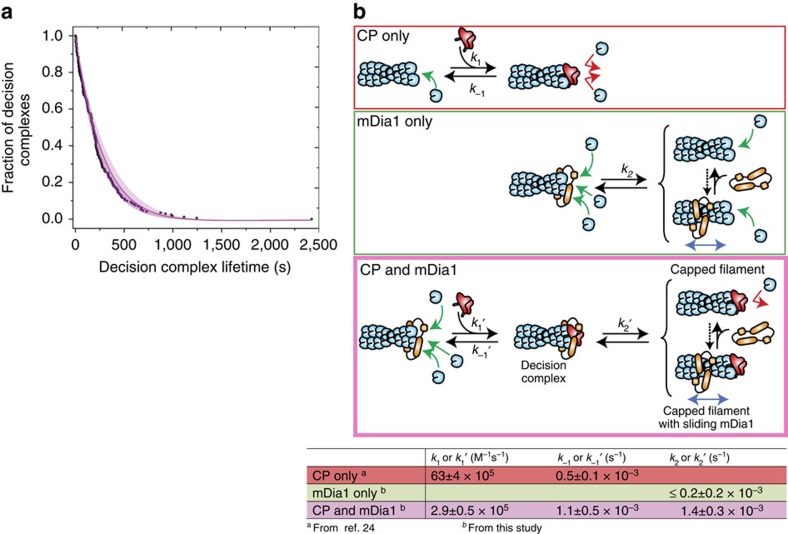
Decision complex kinetics and mechanism. (**a**) Cumulative lifetime distribution of decision complexes containing 549-mDia1 and 649-CP (black; *N*=171), and a maximum likelihood fit (see Methods) of the measured lifetimes to a single-species (exponential) kinetic model (red; shading indicates 95% CI). Fit yielded a characteristic lifetime μ=359 s (s.e.: 41 s; 95% CI: 290–448 s); this quantity includes contributions from both 549-mDia1 and 649-CP dissociation and from photobleaching of both dyes. (**b**) Deduced kinetic schemes and rate constants (±s.e.) for the interaction of actin filament barbed ends with CP only (red box), mDia1 only (green box) or both (magenta box). Green and red arrows mark structures that can or cannot, respectively, add actin monomers to the filament end. Values of *k*_−1_′ and *k*_2_′ are corrected for dye photobleaching (see Methods). The value of *k*_2_ is not corrected for photobleaching; it is an estimated upper limit since only a single 549-mDia1 disappearance event was observed, and this may have been caused by photobleaching rather than dissociation.

To more fully understand the nature of the decision complex, we investigated its kinetic behaviour. The *N*=171 complexes we observed had an exponential lifetime distribution (Fig. 5a). The mean lifetime of the decision complexes that ended by CP dissociation (199±36 s (s.e.m.); *N*=51) was the same within experimental uncertainty as those that ended by mDia1 dissociation (171±24 s; *N*=67). These data are consistent with the hypothesis that the same, single decision complex species produces both outcomes. This model is corroborated by our observations that mDia1 could return to a 649-CP-capped barbed end (thus re-forming the decision complex), wait for 649-CP to dissociate, and then recommence elongation (Fig. 4a,b and Supplementary Movie 6). These observations show that the formation of the decision complex can occur from either direction. Thus, multiple lines of evidence suggest that the decision complex is the single kinetically significant intermediate in the CP/mDia1 antagonism mechanism, can be formed by starting from either a CP-capped, static barbed end or an mDia1-bound, growing barbed end, and can yield either of these barbed-end complexes as an outcome (Fig. 5b, magenta).

The single-molecule measurements directly yield rate constants (Fig. 5b) that predict CP inhibition of mDia1-mediated filament elongation similar to that observed in bulk and that quantitatively define the associative competition process (see Methods). Binding of 649-CP to 549-mDia1-bound ends was >20-fold slower than to barbed ends without formin (*k*_1_′ versus *k*_1_ in Fig. 5b) showing that mDia1 markedly protects barbed ends from CP binding, but does not prevent it altogether. The analysis also shows that CP kinetically destabilizes mDia1 binding to the barbed end: unambiguous dissociation of mDia1 from the decision complex was observed frequently (for example, Fig. 4) and was at least sevenfold faster than its dissociation from a barbed end where mDia1 alone was present (*k*_2_′ versus *k*_2_ in Fig. 5b). Finally, the kinetic analysis predicts that associative competition is likely to be the dominant pathway over dissociative competition. The estimated total concentration of CP in cells is ∼1 μM (ref. 3), although the free concentration may be lower^33^. At this concentration, the rate at which an mDia1 elongating filament would be capped through the associative pathway is calculated to be 0.0014, s^−1^ (see Methods). This is sevenfold or more higher than the rate of capping predicted for the dissociative pathway, which is presumably limited by the rate of spontaneous mDia1 dissociation (*k*_2_ in Fig. 5b).

## Discussion

It has long been established from biochemical and genetic studies that formins and CP have antagonistic activities on actin filament barbed ends^16^,^17^,^18^,^19^,^20^,^26^,^34^. In these studies, it has been assumed that the underlying mechanism is one of dissociative competition (Fig. 3c). Here we made direct, simultaneous single-molecule observations of mDia1 and CP dynamic interactions with barbed ends, revealing the decision complex, a heretofore unknown intermediate that mediates competition for the barbed end by mDia1 and CP. The resultant associative competition mechanism and the role played by formin molecules sliding along the actin filament represent new processes by which cells may regulate formin occupancy of barbed ends and thereby contribute to control of actin filament growth rates, length distributions and barbed-end capture.

One major consequence of the decision complex-mediated filament length regulation mechanism is that it explains a way in which highly processive formins can be attenuated to produce filaments of the lengths seen in cells. *In vitro*, formins by themselves can ride the growing ends of filaments for minutes, leading to the growth of filaments hundreds of micrometers in length^12^,^14^. We show that physiological concentrations of CP could accelerate the dissociation of mDia1 by sevenfold or more through decision complex formation, which would lead to the production of shorter actin filaments as typically observed in cells. Of course, other processes (for example, filament severing) also are likely to play a significant role in decreasing the lengths of actin filaments *in vivo*.

In the previously assumed dissociative competition mechanism, filament length depends only on formin processivity and polymerization rate. The associative competition mechanism establishes a means by which cells can locally tune the lengths of filaments in their actin arrays by controlling the levels of active CP. This is significant because CP is non-uniformly distributed in cells; furthermore, its activity can be locally regulated by phosphoinositides and modulated by specific CP-binding proteins^3^,^33^,^35^,^36^,^37^.

Also of potential significance to our understanding of actin network dynamics *in vivo* is the observation that CP termination of filament growth can be reversed by the action of mDia1 at the barbed end. This provides a possible mechanism by which cells could restart filament growth in response to specific cues. In addition, the ability of the released mDia1 molecule to slide along the filament and to subsequently re-form a decision complex with CP at the barbed end implies a feedback mechanism that responds to filament length: assuming a constant probability of dissociation from the filament side, a shorter filament will have a higher probability of formin recapture at the barbed end. This will lead to preferential resumption of growth by shorter filaments, yielding a more uniform distribution of filament lengths. This is reminiscent of feedback mechanisms previously proposed to control microtubule length by kinesins^38^,^39^.

Of particular significance, the decision complex provides a mechanism by which CP that is anchored at specific cellular locations might capture filament barbed ends being elongated by formins, imposing precise yet dynamic control of filament length and position. In fact, CP and formins colocalize at many of the same cellular structures that capture filament barbed ends (for example, filopodial tips and sarcomeric Z disks)^3^,^5^,^6^,^40^,^41^.

While our observations have defined both the stoichiometry and the dynamic behaviour of the decision complex, its three-dimensional structure has yet to be determined. Formins processively elongate filaments via a stair-stepping mechanism in which the two halves of the formin dimer alternately block both protofilament ends (‘closed' conformations(s)) or only a single protofilament end, leaving the other exposed (‘open' conformation)^2^. For this reason, it is tempting to speculate that the decision complex has a ‘foot in the door' structure in which the formin blocks only one protofilament end (as in the open conformation) and that the other protofilament end is occupied by one of the two CP subunits. This interpretation is consistent with our observations that the decision complex is less kinetically stable than the complexes made by either protein alone with the barbed end.

## Methods

### Protein purification and labelling

mDia1 (ref. 26), SNAP-mDia1 (ref. 14), SNAP-DAAM1 (ref. 31), rabbit muscle actin^42^, CapZ^43^ and human profilin^15^ were purified as described. Both mDia1 and SNAP-mDia1 constructs consist of the FH1, FH2 and C-terminal domains^14^,^26^. Pyrene-labelled^44^ and Alexa Fluor 488-labelled rabbit muscle actin^45^ were prepared as described. SNAP-mDia1 was labelled during purification as described^14^ using SNAP-surface-549 (New England Biolabs, Ipswich, MA) either by itself or together with an analogous benzylguanine derivative of Dyomics 649 (Dyomics, Jena, Germany), yielding 549-mDia1 and 649-mDia1 subunits, respectively. Benzylguanine dye adducts were reacted with protein in 2.5–5.0 adduct:protein mole ratios (15–30 μM adduct). Labelling stoichiometries of the 549-mDia1 and 549-mDia1/649-mDia1 were calculated from the dye absorbance in the labelled protein preparations (using *ɛ*_549_=2.5 × 10^5^ M^−1^ cm^−1^ and *ɛ*_649_=0 for the 549 dye, and *ɛ*_549_=8.5 × 10^3^ M^−1^ cm^−1^ and *ɛ*_649_=1.5 × 10^5^ M^−1^ cm^−1^ for the 649 dye) and protein concentrations measured by densitometry of Coomassie Blue-stained SDS–PAGE gels using BSA as a standard. Measured labelling stoichiometries were 0.79 mol dye per mol protein monomer for 549-mDia1 (ref. 14) and 0.39/0.17 for 549-mDia1/649-mDia1. For storage, labelled formin and capping protein preparations were frozen with liquid N_2_ in HEK buffer (20 mM HEPES (pH 7.5), 1 mM EDTA and 150 mM KCl) supplemented with 1 mg ml^−1^ BSA and 10% glycerol and stored at −80 °C.

### Preparation of 649-CP

A plasmid pSNAP-CAPZ, for simultaneous expression of a His- and SNAP-tagged β1 subunit and untagged α1 subunit of chicken CapZ, was derived from a plasmid for expressing the untagged subunits (pET-3d-βI′/βII in ref. 43) as follows: a DNA insert encoding MHHHHH, followed by the SNAP tag, followed by the linker SGSGSG was prepared by PCR amplification from T7(II)SNAP (New England Biolabs, Ipswich, MA) using primers 5′-GGACCTGGACATATGCACCACCACCACCACCACATGGCTAGCACCATGG-3′ and 5′-GAGCTCGAGCATATGGCTGCCGCTGCCGCTGCCATTAACCTCGAGCCCGGGGG-3′, digested with NdeI, and then ligated to NdeI-digested pET-3d-βI′/βII. Sequencing of the resulting plasmid revealed correct orientation of the insert and a single point mutation in the SNAP tag, L69Q (T7 (II) SNAP numbering), which did not affect labelling.

To express protein, pSNAP-CAPZ was transformed into BL21(DE3)pLysS cells, grown at 37 °C to OD_600_ ∼0.6, induced with 0.4 mM isopropyl-β-D-thiogalactoside, and grown further for 8 h before collecting the cells by centrifugation. Cells were used immediately or frozen in liquid nitrogen. The SNAP-tagged CapZ was purified and labelled with a protocol similar to the one used to purify SNAP-mDia1 (ref. 14), except that the cells were lysed in the presence of 0.5 mg ml^−1^ of lysozyme, and sonicated with a probe sonicator for 3 min on ice. SNAP-CapZ was fluorescently labelled using a procedure analogous to that used to prepare 649-mDia1, except that the labelling reaction used 9 μM (∼4-fold excess) dye adduct for 2 h at room temperature, yielding 649-CP. In-gel fluorometry using the total dye added to the labelling reaction as an internal standard together with the measured protein concentration showed that the labelled protein contained 1.03 mol dye per CP heterodimer. We confirmed that the 649-CP preparation was essentially 100% dye labelled by observing capping protein binding to filaments with TIRF microscopy (Supplementary Fig. 1), which showed nine of nine filaments elongating before 649-CP addition bound a 649-CP at the time of elongation arrest.

### Pyrene–actin assembly assays

Pyrene–actin assembly assays were performed as previously described^20^ with slight modifications. Briefly, rabbit muscle actin preparations were thawed and pre-cleared in a TLA-120.1 rotor (Beckman Coulter, Indianapolis, IN) at 90 000 r.p.m. for 1 h before use. To make samples for the assembly assays, 2 μM 5% pyrene-labelled rabbit muscle actin in G-buffer (3 mM Tris (pH 8.0), 0.2 mM ATP, 0.2 mM CaCl_2_ and 0.5 mM dithiothreitol) was added to 10 × exchange buffer (1 mM MgCl_2_ and 10 mM EGTA), then to either HEK buffer, or proteins in HEK buffer (2 nM mDia1 and 5 μM profilin). This reaction was allowed to incubate for 2 min, then mixed with 20 × initiation mix (40 mM MgCl_2_, 10 mM ATP and 1 M KCl) for a final volume of 58 μl and pipetted into a quartz microcuvette. Data were acquired on a PTI fluorometer (Photon Technologies International, Lawrenceville, NJ) at 25 °C, *λ*_ex_=365 nm, *λ*_em_=407 nm. At ∼100 s, acquisition was paused and either 2 μl HEK buffer or the indicated concentration of CP was added to the reaction. Curve before the addition of CP (Fig. 1a, black) is the average of all of the pre-CP addition traces. Post-CP intensity data were offset to start at a common point.

To find the half-maximal inhibitory concentration (IC_50_) from the experimental pyrene–actin assembly data, assembly rates before and after the addition were measured by linear regression of the data record segments from ∼60–100 and ∼130–170 s, respectively. Errors in the individual points (Fig. 1b) reflect the s.e. from the linear regression analysis. The ratios of these slopes were plotted and fit according to


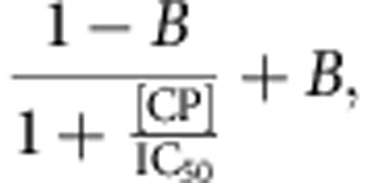


where [CP] is the capping protein concentration and IC_50_ and *B* are fit parameters. To determine the IC_50_ predicted by the associative competition kinetic model derived from the single-molecule fluorescence measurements, simulated pyrene fluorescence curves were constructed by numerical integration of the rate equations (assuming a negligible concentration of free mDia1) and analysed using the same procedure as for the experimental pyrene fluorescence records. The simulations yielded IC_50_=8.8±3.6 nM, indistinguishable from the experimental value IC_50_=10±4 nM (Fig. 1b).

### TIRF microscopy

TIRF imaging was performed on a custom-built multi-wavelength micromirror microscope as described^21^,^45^, with the following minor changes to the slide passivation protocol, reagents and image acquisition protocol. Briefly, slides were passivated with polyethylene glycol–silane (PEG-silane; 2 kDa, 2–4 mg ml^−1^, Laysan Bio, Arab, AL) by reacting the microscope slide overnight with the PEG–silane in 80% ethanol, pH 2 at 70 °C. TIRF buffer was prepared as a stock at 2 × the final composition of 10 mM imidazole (pH 7.2), 50 mM CH_3_COOK, 1 mM MgCl_2_, 1 mM EGTA, 10 mM dithiothreitol, 0.2 mM ATP, 15 mM glucose, 0.002 mg ml^−1^ catalase, 0.01 mg ml^−1^ glucose oxidase, 0.1% BSA (Calbiochem, (Merck) Darmstadt, Germany) and 3–5% 200 kDa dextran. Proteins were individually diluted in G-buffer supplemented with 1 mg ml^−1^ BSA, then mixed with the 2 × TIRF buffer and water so that the indicated final protein concentrations were in 1 × TIRF buffer supplemented with 0.5 × G-buffer. Ten per cent AF488-labelled actin (0.5–1 μM, final) was combined with a threefold excess of profilin, 0–100 pM of 549-mDia1 or 549-DAAM1, 0–12 nM CP or 649-CP, and TIRF buffer, quickly mixed, then introduced into a 15-μl flow cell. Except where otherwise indicated, two images of 0.5 s duration were collected every 5 s, one with 488 nm excitation (75 μW incident to micromirror) and the other with simultaneous 532 and 633 nm (each 600 μW incident) excitation. Separate sub-images were formed from emissions <633 nm and >633 nm (ref. 21). One pixel was equivalent to 122 × 122 nm. Experiments were limited to sub-nanomolar concentrations of formins to avoid nucleation of excessive numbers of filaments.

### Image processing

Images recorded at different wavelengths were aligned as described^45^,^46^, converted into RGB AVI files, and imported into ImageJ (NIH, Bethesda, MD). Contrast and brightness were adjusted independently for each colour. Each colour channel of images displayed in figures and Supplementary Movies was spatial filtered to suppress noise (10 pixel rolling ball subtraction, then 3 × 3 pixel median filter). Time records of fluorescence intensities from filament ends were computed from each unfiltered image as the average intensity in the 3 × 3 pixel square centred at the end of the filament minus the average intensity in a nearby 3 × 3 pixel background area.

### Single-molecule kinetic analysis

Times at which 649-CP and 549-mDia1 bound to filament ends were tabulated by inspection of unfiltered movies. Apparent first-order rate constants for association of 649-CP to 549-mDia1-bound filament ends at various 649-CP concentrations were calculated as the number of 649-CP-binding events divided by the total time a barbed end occupied only by 549-mDia1 was visible. A proportional fit of the apparent first-order rate constants versus concentration (weighted by the inverse square of the standard error) yielded the second-order rate constant *k*_1_′. The association rate constant of CP to filament barbed ends not bound by formin was measured under similar conditions in ref. 24.

In the absence of CP, only a single 549-mDia1 dissociation event was observed in 5,420 s total observation time over 28 filaments, and even the single event may have been due to 549-mDia1 photobleaching (which may occur at a rate of ∼10^−3^ s^−1^; see Supplementary Fig. 4a) rather than dissociation. Therefore, the reciprocal of the 5,400 s observation time was used as the estimate the upper limit of the dissociation rate constant for mDia1 (*k*_2_).

To calculate the characteristic lifetime of the 649-CP/mDia1/filament end complex, we measured the lifetime *τ* of each complex until it dissociated by loss of 649-CP or 549-mDia1. Control experiments (Supplementary Fig. 4) showed that photobleaching did not substantially reduce the lifetime under the conditions used. For complexes that were not observed to dissociate, we separately compiled the time *n* that each was observed until observation was truncated by termination of the video recording, the filament end leaving the microscope field of view or observation of the filament end being blocked by the growth of other filaments. In analysing these data, we used a modified version of the approach described in ref. 47. Specifically, the set of individual measured lifetimes and truncated observation durations were globally fit using the maximum likelihood algorithm to the probability density functions


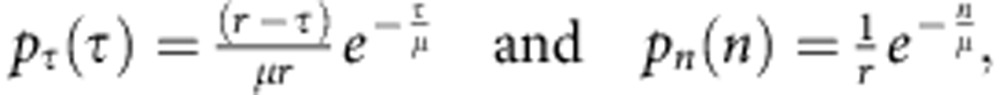


respectively, where *r* is the mean recording length weighted by the number of complexes observed in each recording and the fit parameter *μ* is the characteristic lifetime. Derivation of these functions is based on the approximation that *τ* is significantly smaller than *r*. This condition is satisfied in our data sets, where every measured *τ* was less than *r* and the (*τ*/*r*) distribution across all five experimental conditions (Supplementary Fig. 4) had median 0.1 and 90th percentile 0.33. The analytical method and its software implementation were validated using data produced by numerical simulations designed to mimic the effects of experimental data truncation. The confidence intervals for the fit parameter and fit curves were estimated by bootstrapping. The reported mean lifetimes for complexes that terminated by 649-CP or 549-mDia1 are less than *μ* because the former do not include the truncated observations (53 of 171 total).

The rate constants for dissociation of the paused filament complex (Fig. 5b, magenta) were calculated as


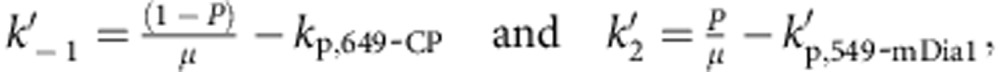


where *k*′_p,549-mDia1_ and *k*_p,649-CP_ are the measured photobleaching rates and *P*=0.568±0.004 was the fraction (±s.e.) of complexes observed to end by loss of 549-mDia1 fluorescence rather than by loss of 649-CP fluorescence. *k*_p,649-CP_ was calculated as described (Supplementary Fig. 4); *k*′_p,549-mDia1_ was taken to be the weighted mean of *k*_p,549-mDia1_ (Supplementary Fig. 4) and *k*_2_ (Fig. 5b) since the latter provides an independently measured upper limit on the 549-mDia1 photobleaching rate.

All reported uncertainties in lifetimes and rate constants are standard errors.

The overall flux *R* through the associative competition pathway (Fig. 3c, bottom) expected at a given concentration of capping protein [CP] was calculated as


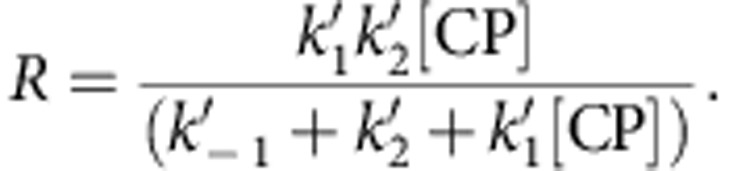


## Additional information

**How to cite this article:** Bombardier, J. P. *et al*. Single-molecule visualization of a formin-capping protein ‘decision complex' at the actin filament barbed end. *Nat. Commun.* 6:8707 doi: 10.1038/ncomms9707 (2015).

## Supplementary Material

Supplementary Figures and Supplementary Reference Supplementary Figures 1-5 and Supplementary Reference

Supplementary Movie 1 Actin filament elongation by one- and two-color-labeled mDia1. Three-color TIRF showing actin filaments (blue) being processively elongated by individual mDia1 molecules labeled with 549 dye only (yellow), 649 dye only (red), or both 549 and 649 dyes (yellow and red), as in Fig. 2a. Diameter of the circular field of view: 61 μm. Playback speed: 31×.

Supplementary Movie 2 Processive actin filament elongation by mDia1. Movie shows 549-mDia1 (yellow) processively elongating the barbed ends of actin filaments (blue) in the absence of CP. Field diameter: 60 μm. Playback speed: 31×.

Supplementary Movie 3 Example 1: Transition at a barbed end from mDia1-mediated growth to decision complex to CP capped. 549-mDia1 (yellow) mediated actin filament (blue) barbed end elongation, indicated by a green arrowhead, was terminated by the binding of 649-CP (red) and the formation of a kinetically stable 'decision complex' (magenta arrowhead) that subsequently yielded a 649-CP-capped filament (red arrowhead) after dissociation of 549-mDia1 (corresponds to Fig. 3a,b). Image size: 13 × 41 μm. Playback speed: 31×.

Supplementary Movie 4 Example 2: Transition at a barbed end from mDia1-mediated growth to decision complex to CP capped. 549-mDia1 (yellow) mediated actin filament (blue) barbed end elongation, indicated by a green arrowhead, was terminated by the binding of 649-CP (red) and the formation of a kinetically stable 'decision complex' (magenta arrowhead) that subsequently yielded a 649-CP-capped filament (red arrowhead) after dissociation of 549-mDia1. Image size: 17 x 23 μm. Playback speed: 31×.

Supplementary Movie 5 Decision complex formation is reversible. 549-mDia1 (yellow) mediated elongation of an actin filament (blue), indicated by a green arrowhead, was interrupted twice by the binding of 649-CP (red), resulting in the formation of a kinetically stable 'decision complex' (magenta arrowhead). The first time, the decision complex resolved by 649-CP dissociation from the complex and resumption of 549-mDia1-mediated filament elongation. The second time, 549-mDia1 slid away from the barbed end and then dissociated from the filament, yielding a 649-CPcapped filament (red arrowhead). Image size: 16 × 26 μm. Playback speed: 31×. Corresponds to Supplementary Figure 5.

Supplementary Movie 6 Sliding along an actin filament of a 649-mDia1 molecule and its recapture by the barbed end. Filament end marked by arrowhead is the one shown in Fig. 4a,b (see figure legend). Image size: 13 × 30 μm. Playback speed: 31×.
