# Supplementary material for: Single-molecule visualization of a formin-capping protein ‘decision complex' at the actin filament barbed end
Source: Nat Commun. 2015 Nov 13;6:8707. doi: 10.1038/ncomms9707 (PMC4660045; doi:10.1038/ncomms9707)
Supplement: Supplementary Figures and Supplementary Reference — Supplementary Figures 1-5 and Supplementary Reference [file ncomms9707-s1.pdf]

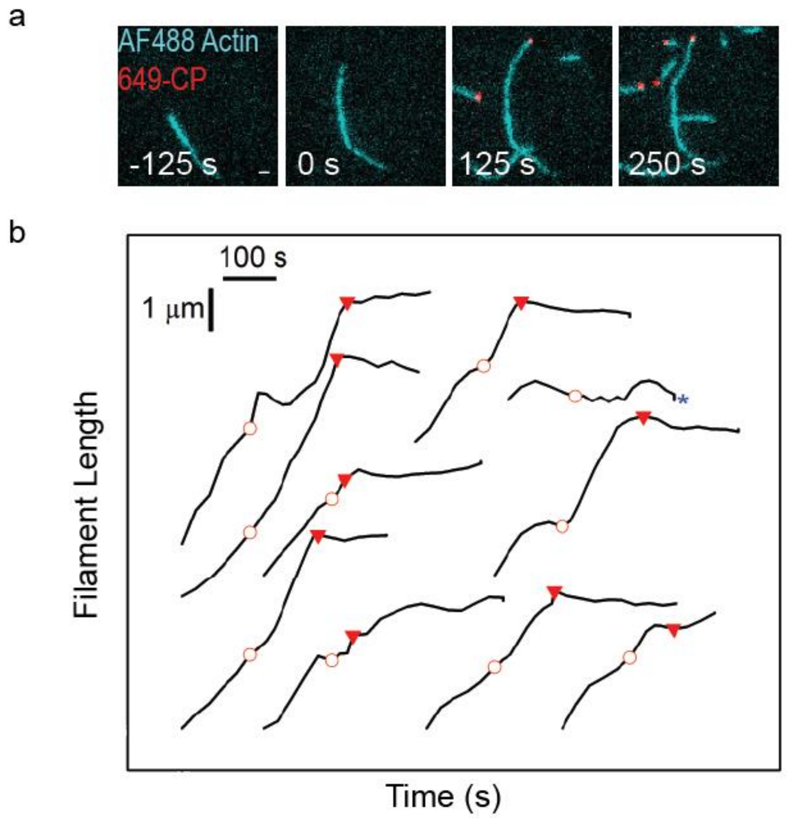

Supplementary Figure 1. In the absence of mDia1, 649-CP quickly arrested actin filament elongation by binding to barbed ends (*N* = 9 binding events observed). (**a**) Time sequence of color-merged TIRF microscopy images of a single area of interest. Actin filaments (cyan) were allowed to grow from −125 s to 0 s, then 1.5 nM 649-CP (red) was added into the flow cell. Bar: 1 µm. (**b**) Length records (offset for clarity) of ten filaments measured at 5 s intervals before and after 649-CP addition. Records were concatenated at the time of 649-CP addition (circles) and the time at which 649-CP appeared at the end is indicated (triangles). Nine filaments stopped elongating when 649-CP bound; one (star) stopped spontaneously before 649-CP was introduced.

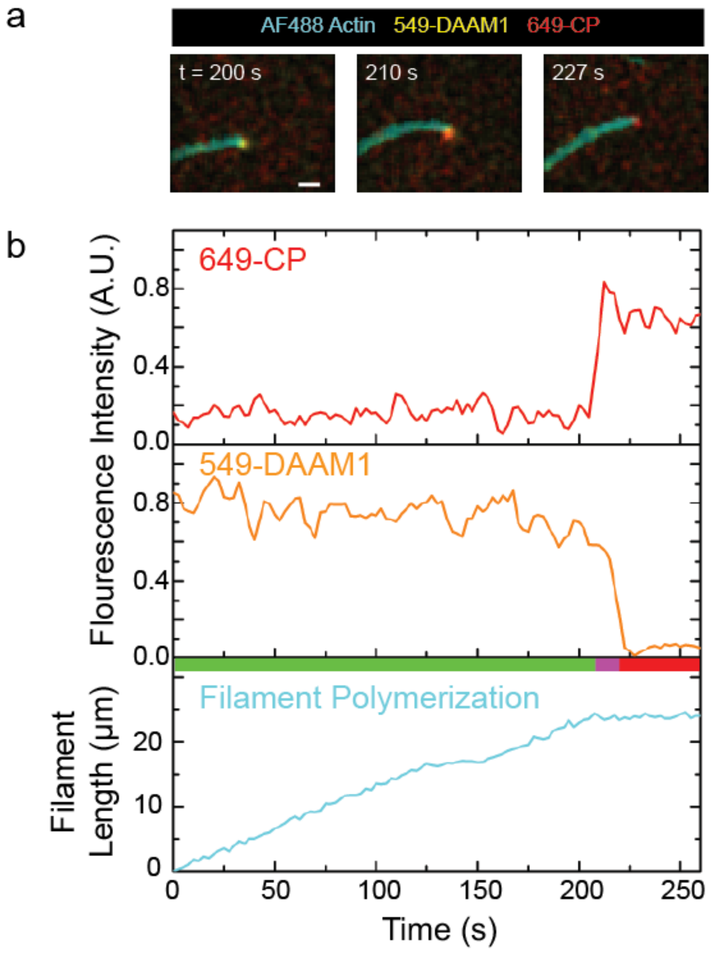

Supplementary Figure 2. Example of a DAAM1 / CP / barbed end complex. **(a)** Merged three-color TIRF image of the actin filament (blue) at indicated times before the formation of the barbed end complex (only 549-DAAM1 at the barbed end, 200 s), when 549-DAAM1 (yellow) and 649-CP (red) molecules were bound simultaneously to the barbed end (210 s), and after the 549-DAAM1 had dissociated from the barbed end (227 s). 549-DAAM1 is a green-dye-labeled analog of the SNAP-649-CDaam1 described previously[1]. **(b)** Fluorescence intensity and filament length record of the filament in (A). Color ribbon indicates the time intervals in which the barbed end is occupied by 549-mDia1 only (green) 640-CP only (red), or both proteins (magenta). Scale bars: 2 µm. Frame interval: 2.5 s. **Population data:** On 10 of 30 filament ends, we observed colocalization of 649-CP/549-DAAM1. These DAAM1-CP decision complexes (e.g., that in (a) and (b)) appeared roughly ten-fold shorter lived than the mDia1-CP decision complex; this could reflect faster dissociation of the complex or lower photostability of the 549-DAAM1 construct. However, as observed for mDia1, CP clearly caused 549-DAAM1 dissociation from barbed ends: 549-DAAM1 dissociation from filaments ends in the absence of CP was almost never seen. In contrast, the majority of filaments observed in a sample with both 549-DAAM1 and 649-CP were seen to become capped with only 649-CP at the barbed end during the ~650 s observation period. Taken together, these results suggest that just as it does for mDia1, CP displaces DAAM1 by the formation of a decision complex in which both proteins are bound simultaneously to the filament barbed end.

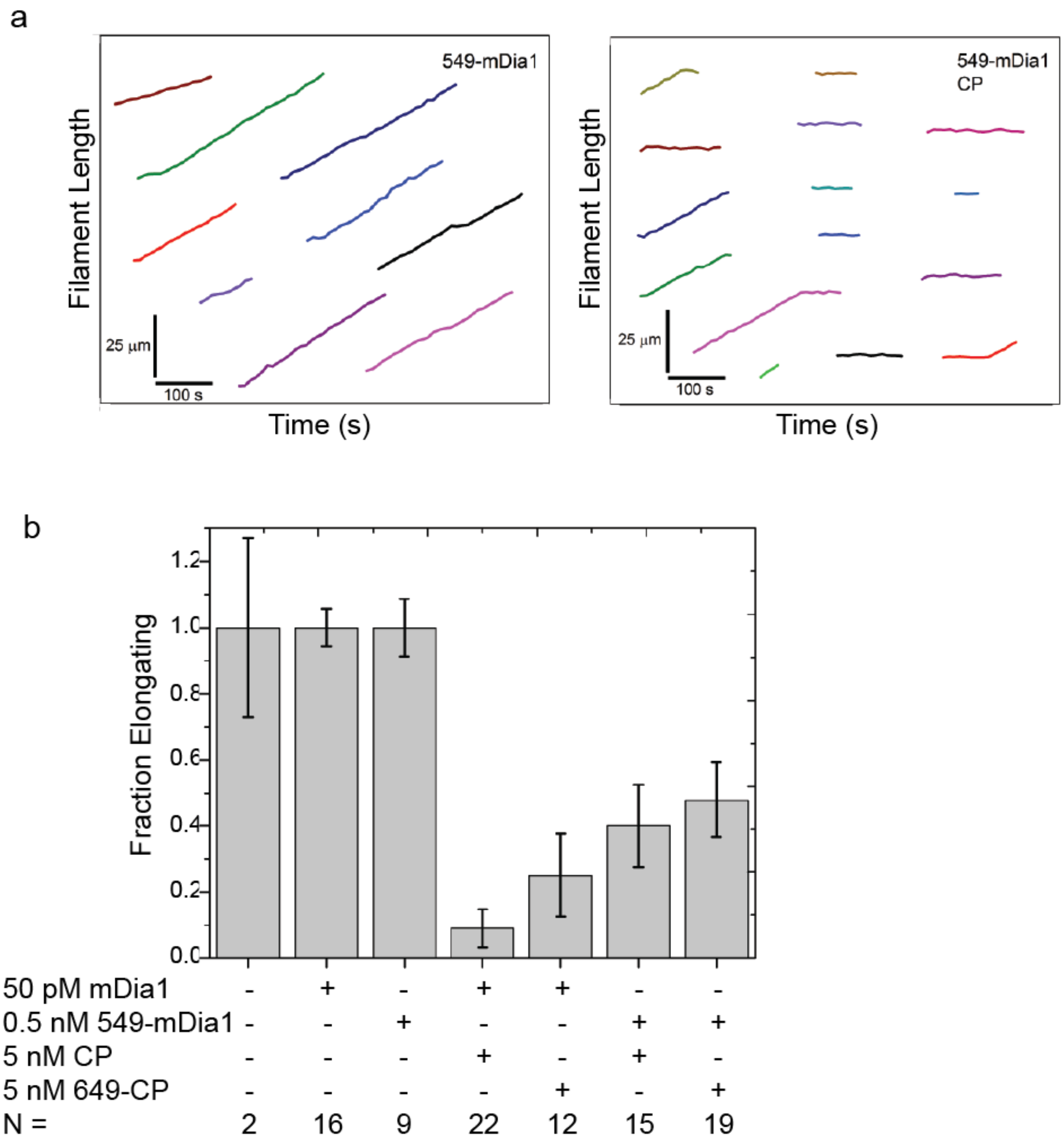

Supplementary Figure 3. Inhibition of mDia1-mediated filament elongation by CP was not affected by tagging and labeling the proteins. Actin (0.5 µM) was nucleated and polymerized by 549-mDia1 (0.05 nM) or mDia1 (0.5 nM) in the presence or absence of 5 nM of the indicated CP construct and 1.5 µM profilin. (**a**) Length records (offset and multicolored for clarity) of single filaments with 549-mDia1 complexed barbed ends in the absence (left) and presence (right) of untagged CP. (**b**) Fraction (± s.e.) of filaments with growth velocity that fell below a set threshold (0.002 µm s^{-1} at five consecutive time points) during recording. Length records were 50 - 350 s duration. 5 nM 649-CP and 5 nM untagged CP are approximately equally effective (within experimental uncertainty) at stopping filament growth in mDia1+CP mixtures. The same is true when 549-mDia1 and mDia1 are compared. Since the labeled CP proteins stop growth almost exclusively by formation of the associative mDia1 / CP / barbed end complex, the quantitative agreement with the results obtained with unlabeled proteins suggest that the latter stop filament growth via the same complex.

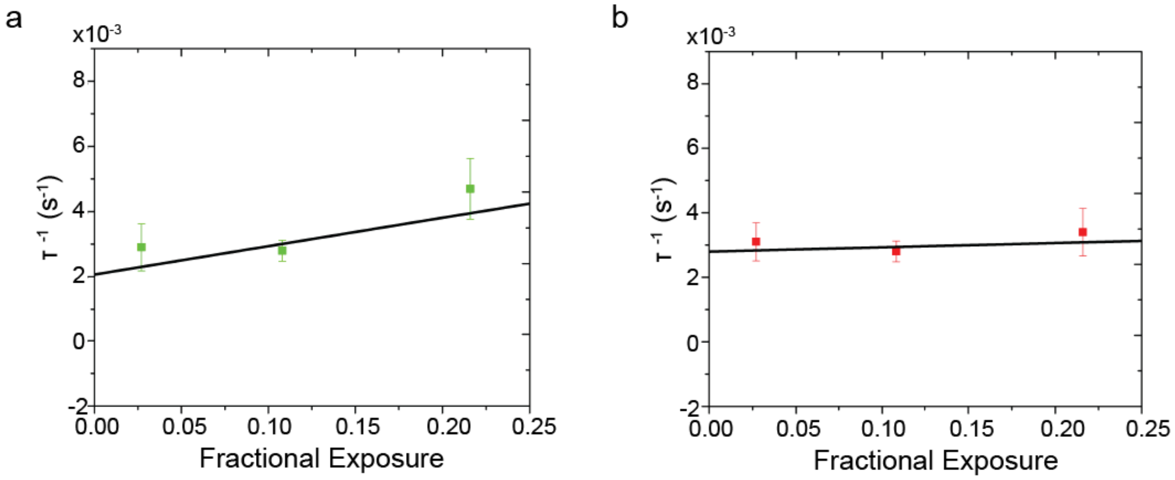

Supplementary Figure 4. Disappearance of the mDia1 / CP / filament barbed end complex was largely or entirely caused by dissociation, not photobleaching. Points (± s.e.) show the reciprocal of the observed complex lifetime τ (see Methods) as a function of changes to the amount of exposure to the 532 nm (a) or 633 nm (b) TIRF microscope lasers. Fractional exposure is the time that the field of view was being excited by the 532 nm or 633 nm laser, divided by the time from the beginning of one exposure for that laser to the beginning of the next. Fractional exposure was varied by making 0.54 s exposures at intervals of 2.5, 5.0, and 20.0 s. *N* = 38, 171, and 38, respectively, in (a), and 28, 171 (same data as (a)), and 38 in (b). Weighted linear fits (lines) yield slope and intercept (± s.e.) of 0.008 ± 0.008 s^{-1} and 0.0021 ± .0009 s^{-1} in (a); and 0.001 ± 0.004 s^{-1} and 0.0028 ± 0.0004 s^{-1} in (b). The products of the fractional exposure used in the kinetics experiments, 0.11, and the slopes give the estimated photobleaching rates $k_{p,549\text{-mDia1}}$ = 0.0009 ± 0.0009 s^{-1} (a) and $k_{p,649\text{-CP}}$ = 0.0001 ± 0.0004 s^{-1} (b). Thus, there is no persuasive evidence that the photobleaching rates are significantly greater than zero and they are estimated to be small (a) or negligible (b) in comparison to the decision complex decay rate (Fig. 5a) of 1 / 359 s = 0.0028 s^{-1}.

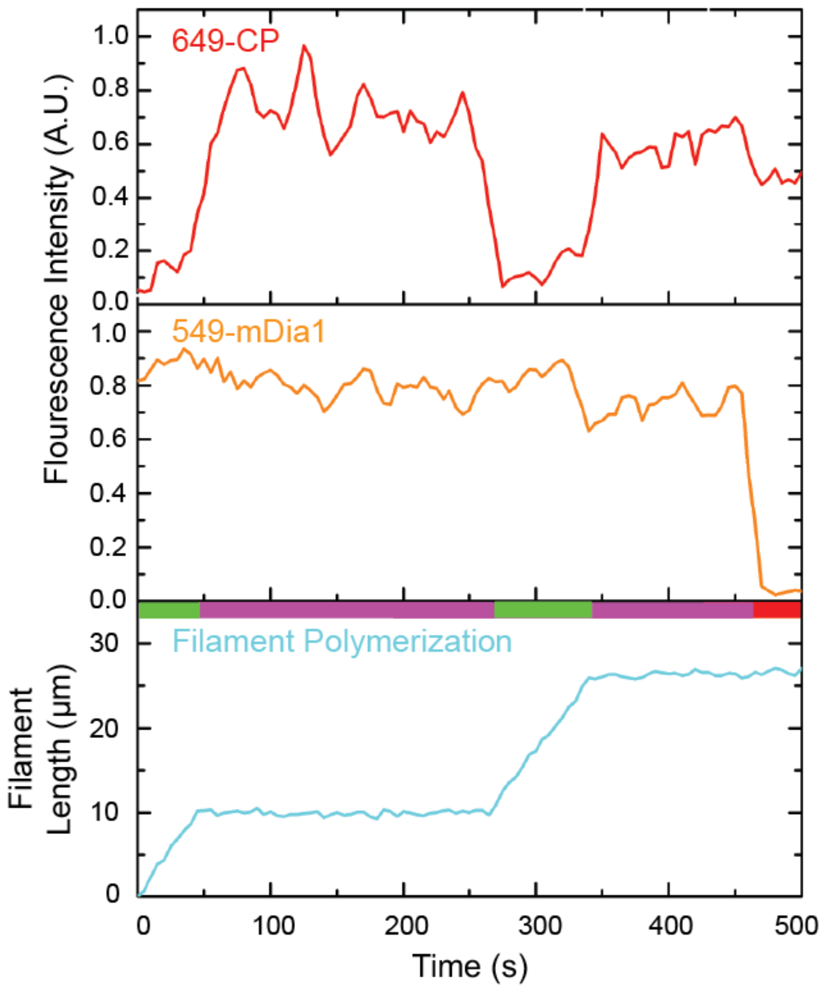

Supplementary Figure 5. The loss of 649-CP from the mDia1 / CP / barbed end complex occurs in a single fluorescence intensity step (*N* = 40 649-CP fluorescence loss events measureable). The plot shows the fluorescence intensities for 649-CP (top) and 549-mDia1 (middle) at the barbed end of a single filament, as well as the length record for the filament. Abrupt 649-CP fluorescence decrease is accompanied by resumption of elongation (at 265 s). Color ribbon indicates the time intervals in which the barbed end is occupied by 549-mDia1 only (green) 640-CP only (red), or both proteins (magenta).

Supplementary Reference

1. Jaiswal, R. *et al.* The formin Daam1 and fascin directly collaborate to promote filopodia formation. *Curr. Biol.* 23, 1373–1379 (2013).
